# Touch engages visual spatial contextual processing

**DOI:** 10.1038/s41598-018-34810-z

**Published:** 2018-11-09

**Authors:** Alexis Pérez-Bellido, Ryan D. Pappal, Jeffrey M. Yau

**Affiliations:** 1 Department of Neuroscience, Baylor College of Medicine, Houston, One Baylor Plaza, Houston, Texas 77030 USA; 2 Present Address: Radboud University, Donders Institute for Brain, Cognition and Behavior, Nijmegen, The Netherlands; 3 Present Address: Washington University School of Medicine in St. Louis, St. Louis, MO 63108 USA

## Abstract

The spatial context in which we view a visual stimulus strongly determines how we perceive the stimulus. In the visual tilt illusion, the perceived orientation of a visual grating is affected by the orientation signals in its surrounding context. Conceivably, the spatial context in which a visual grating is perceived can be defined by interactive multisensory information rather than visual signals alone. Here, we tested the hypothesis that tactile signals engage the neural mechanisms supporting visual contextual modulation. Because tactile signals also convey orientation information and touch can selectively interact with visual orientation perception, we predicted that tactile signals would modulate the visual tilt illusion. We applied a bias-free method to measure the tilt illusion while testing visual-only, tactile-only or visuo-tactile contextual surrounds. We found that a tactile context can influence visual tilt perception. Moreover, combining visual and tactile orientation information in the surround results in a larger tilt illusion relative to the illusion achieved with the visual-only surround. These results demonstrate that the visual tilt illusion is subject to multisensory influences and imply that non-visual signals access the neural circuits whose computations underlie the contextual modulation of vision.

## Introduction

How we perceive a stimulus depends strongly on the spatial context in which the information is experienced. Contextual modulation has been studied extensively in vision^1,2^, where it has been shown that the perception of color, orientation, contrast, and motion changes as a function of the context in which these features appear^3–5^. Substantial progress has also been made in understanding the neural computations underlying visual spatial context processing^5,6^. However, we rarely experience environmental context through only one sensory modality; our typical perceptual experience involves integrating information from multiple senses. Multisensory integration is useful because the processing of redundant sensory cues can serve to increase signal reliability and resolve ambiguities^7,8^. Given the brain’s tendencies to combine multisensory cues, we speculated that interactions between sensory modalities could also shape the patterns of contextual modulation that are typically studied from a unisensory perspective. Establishing that signals in one modality influence contextual processing of signals in another modality would inform our understanding of the neural circuits involved in multisensory processing.

We perceive spatial information by vision and touch^9,10^. Because objects are frequently seen and touched simultaneously, neural representations of visual and tactile spatial information might be analogous and/or shared at multiple levels of processing^11–14^ and these representations likely support perceptual interactions between vision and touch^15–19^. Such highly-specific interactions in feature processing may emerge in part from more general spatial attention interactions between the visual and somatosensory systems^20–22^. These crossmodal links in spatial attention, in addition to finely tuned tactile influences on binocular visual processing^8,17^, imply that touch engages in the cortical circuits mediating low-level visual processing. Because a variety of visual contextual modulation effects are also thought to emerge from neural computations performed in low-level visual processing, we reasoned that tactile signals could also modulate the visual spatial context processing.

**Figure 1:**
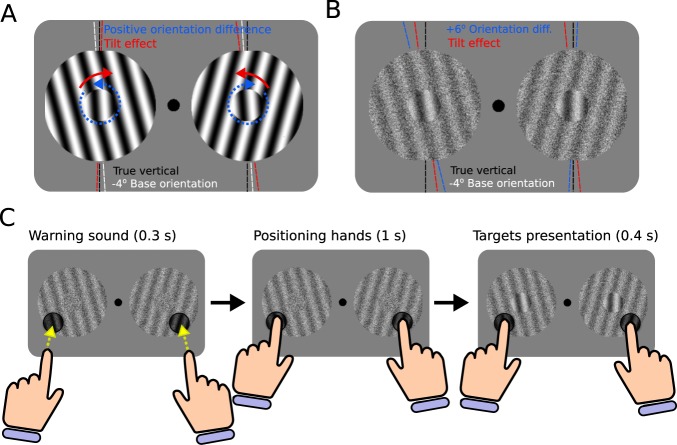
Experimental design (**A)** For illustrative purposes, a non-noisy version of the −4° base orientation with 0° orientation difference condition (both central gratings lean −4° towards the left). The contextual surrounds repel both central gratings inwards (red marks), making the left central grating to look more vertical than the right one. A positive orientation difference (blue arrows) would be required to counteract the tilt illusion and make both central gratings to look equally vertical (black vertical lines). (**B**) Combining a −4° base orientation with +6° orientation difference condition (blue lines) makes the orientation of the right central grating appear closer to the vertical than the left central grating (black lines). This difference is predominantly indexed by the asymptotic lapse parameters (λ in this particular example). The tilt illusion reduces the difference in verticality between both central gratings by pulling the left central grating towards the vertical and pushing the right central grating away from the vertical (red lines). (**C**) Example of a bimodal visuo-tactile (VT) trial sequence. Participants waited to hear the low-frequency tone to touch the tactile stimuli (depicted by the dark circles) overlaid on the visual surround with their index fingers. Then after 1.3 s, the two central gratings were briefly added to the presentation for 0.4 s. Finally, a high-frequency tone signaled the participants to remove their fingers from the tactile stimuli and to respond using a foot pedal which of the two central gratings was more closely oriented to the (subjective) vertical (for illustrative purposes, the contrast of the surrounds in this figure have been enhanced with respect to the contrast levels tested in the actual experiment).

To probe for tactile influences on visual contextual processing, we exploited the visual tilt illusion^5,23^, a well studied phenomenon in which the perceived orientation of a central grating is modulated by the orientation of a grating presented in its surround. Specifically, the central grating appears to be shifted away from that of its surround (~3° repulsive effect) when their orientation difference is small (e.g., 10°–20°) and shifted toward the orientation of the surround (~0.5° attractive effect) when their difference is large (e.g., 70°–80°)^23^. Given our hypothesis that tactile signals engage in the neural circuits mediating visual contextual modulation, we predicted that an oriented tactile grating touched in a region surrounding a visual grating would bias orientation discrimination of the visual grating in a manner consistent with the visual tilt illusion. We tested this prediction using a bias-free paradigm (Fig. 1) under two different experimental manipulations (Methods). First, we tested whether a tactile surround alone is sufficient to induce the visual tilt illusion. We predicted that if tactile orientation information is systematically mapped in a spatially-specific manner to visual cortex, touching a contextual haptic grating should suffice to induce the visual tilt illusion. Second, we tested whether multisensory integration of visual and tactile orientation signals in the surround, which may enhance the strength and reliability of orientation information in the surround^19^, can modulate the magnitude of the tilt illusion. We predicted that the strength of the induced tilt illusion should increase under the bimodal compared to the visual-only condition if combining visual and tactile oriented surrounds improves the sensory representation of the surround.

## Results

### Exploratory analyses

We first conducted separate two way repeated-measures ANOVA on estimates of the point of subjective equality (PSE, a measure of bias), the just-noticeable difference (JND, a measure of sensitivity), and parameters corresponding to lapse rates (λ and γ) with surround condition (None (N), Tactile-only (T), Visual-only (V), Visuo-tactile (VT)) and base orientation (−4°, 4°) as the within-subjects factors. The rmANOVA on the PSE showed a significant main effect of surround condition (F_3,51_ = 30.43, P < 0.001, η = 0.247), but neither the main effect of base orientation (F_1,17_ = 0.4, P = 0.053) nor the surround condition × base orientation interaction (F_3,51_ = 1.68, P = 0.181) achieved statistical significance. The rmANOVA on the upper bound λ parameter also showed only a significant main effect of surround condition (F_3,51_ = 7.1, P < 0.005, η = 0.086). There were no significant main or interaction effects for the JND and lower bound γ parameters. Because performance did not differ meaningfully between the two base orientation manipulations, we collapsed the data over the base orientations to reduce the parameter space in subsequent analyses.

**Figure 2:**
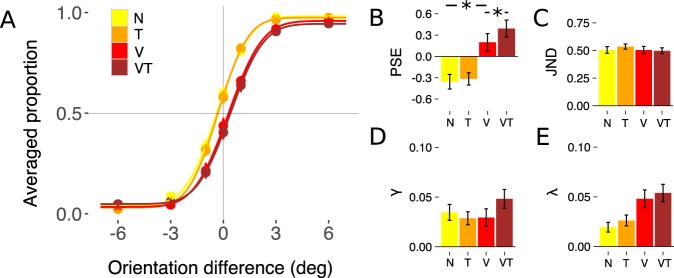
Orientation discrimination performance (**A**) Group averaged choice probability data and group level psychometric functions for each surround condition (None, Tactile-only, Visual-only and Visuo-tactile). Mean of the group distribution of individual PSE (**B**) JND (**C**) γ (**D**) and λ (**E**) parameters. Error bars represent standard error. Asterisks represent those hypothesis-driven contrasts that showed a significant effect.

Although we predicted PSE changes *a priori*, we did not have clear predictions on how manipulations of the surround would impact estimates of λ. Thus, given the significant main effect of surround condition on estimates of λ (Fig. 2E), we performed post-hoc paired t-tests on this lapse parameter. These analyses showed that the λ parameter was larger in the Visual-only and Visuo-tactile conditions compared to Tactile-only condition (λ_V_ vs λ_T_: t_(17)_ = 2.28, P < 0.04; λ_VT_ vs λ_T_: t_(17)_ = 3.23, P < 0.005) and the condition without a surround (λ_V_ vs λ_N_: t_(17)_ = 2.49, P < 0.04; λ_VT_ vs λ_N_: t_(17)_ = 3.72, P < 0.005).

### Hypothesis-driven analyses

The exploratory analyses revealed that PSE estimates differed across surround conditions. To better understand the visual and tactile surround effects on visual tilt perception, we conducted a rmANOVA on PSE values, organizing the four experimental conditions into two factors, to explore main and interaction effects of visual surround (presence vs absence) and tactile surround (presence vs absence). We found significant main effects of visual surround (F_1,17_ = 39.2, P < 0.001, η = 0.33) and tactile surround (F_1,17_ = 4.5, P = 0.047, η = 0.017), but the interaction failed to achieve significance (F_1,17_ = 1.4, P < 0.25). These results indicate that visual and tactile surround signals both modulate visual tilt perception. Building on these results, we performed planned comparisons on the PSE estimates to test our more specific *a priori* predictions on the influence of touch on the visual tilt illusion.

### Perception of the central grating with visual surround

**Figure 3:**
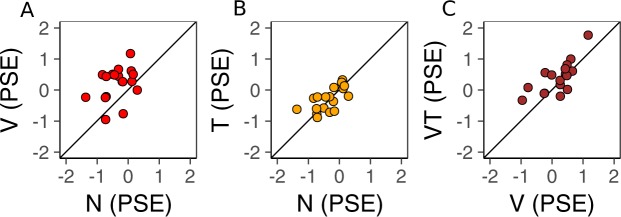
Planned comparisons on PSE values Each point corresponds to PSE estimates for a single participant. (**A**) Comparison between the baseline condition (N) containing no surround orientation information and the visual-only condition (V). (**B**) Comparison between the baseline condition and the tactile-only condition (T). (**C)** Comparison between the visual-only condition and the visuo-tactile condition (VT). Points above the unity line indicate subjects exhibiting larger repulsive tilt illusion effects in the condition plotted on the ordinate compared to the abscissa.

To assess if the visual-only surrounds modulated the perception of the central gratings to induce a visual tilt illusion, we tested whether the PSE in the Visual-only condition was significantly larger than the PSE estimated in the condition in which there was no surround (Fig. 3A). On average, PSE_V_ exceeded PSE_N_ by 0.55° and this difference was significant according to a one tailed paired t-test (t_(17)_ = 4.32, P < 0.001, Cohen’s d = 1.29; PSE_V_ > PSE_N_ in 15/18 subjects). This result demonstrates that the visual surrounds effectively repulsed the perceive orientation of both central gratings, such that central grating marked by substantial orientation differences (indexed by the PSE) were perceived to be identical. This pattern is consistent with previous reports of the visual tilt illusion.

### Perception of the central grating with tactile surround

To assess if tactile surrounds alone could modulate the perception of the central gratings, we tested whether the PSE in the Tactile-only condition was significantly larger than the PSE estimated in the condition in which there was no surround (Fig. 3B). Although PSE_T_ was qualitatively larger than PSE_N_ on average (∆PSE = 0.04°), this difference was not statistically significant according to a one tailed paired t-test (t_(17)_ = 0.49, P = 0.31). These results indicate that simply touching a grating pattern positioned adjacent to a visual grating is insufficient to significantly modulate the perceived orientation of the visual grating.

### Perception of the central grating with visuo-tactile surround

To assess if the addition of redundant tactile information to a visual surround induces larger visual tilt illusions than the visual surround alone, we tested whether the PSE in the Visuo-tactile condition was larger than the PSE in the Visual-only condition (Fig. 3C). On average, PSE_VT_ exceeded PSE_V_ by 0.19° and this difference was significant according to a one tailed paired t-test (t_(17)_ = 2.18, P = 0.021, Cohen’s d = 0.38; PSE_VT_ > PSE_V_ in 13/18 subjects). This result shows that surrounds defined by congruent visual and tactile gratings induced larger repulsive biases in the visual tilt illusion compared to visual surrounds alone.

## Discussion

We investigated whether tactile information can influence the contextual modulation of visual orientation signals. Using a bias-free visual tilt illusion paradigm, we measured how participants perceived the relative orientation of visual gratings presented in the absence of surround information or in the presence of V, T and VT surrounds. We replicated the visual tilt illusion by showing that the perceived orientation of a central grating was repulsed by a surround grating with a similar orientation. While tactile gratings alone did not induce contextual modulation of the central visual grating, a combination of tactile and visual surrounds resulted in a larger tilt illusion than that achieved with only the visual surround. Given an absence of significant interaction effects between the visual and tactile surrounds, the amplified tilt illusion achieved under the VT condition likely reflects a combination of independent visual and tactile surround effects rather than a tactile modulation of visual surround influences. These results indicate that tactile orientation signals engage with the neural circuits involved in visual spatial context processing.

We used a bias-free paradigm to measure the tilt illusion^24,25^ in which observers performed rapid comparisons of two simultaneously presented visual gratings. Moreover, all of the orientation conditions were symmetrically counterbalanced relative to the vertical orientation. Lastly, participants had to use both hands during each trial to explore a flat (control) or ridged tactile stimulus thereby controlling for the motor demands of the task. Together, these design aspects minimize the effects of possible post-perceptual biases in orientation encoding^26,27^ and inoculate our results from concerns that the systematic contextual modulation patterns we observed were merely the result of response biases and task demands. Notably, subjects may not have been entirely unbiased in comparing the central gratings presented on each trial, even in the absence of any surrounds. Indeed, we observed that PSE values in the N and T conditions were significantly smaller than 0 (p < 0.01; paired t-test), even though we would have predicted that PSE values in the N condition should equal 0. This pattern of bias indicates a tendency for participants to report the left central grating as more vertical than the right one when both central gratings are matched in orientation in the −4° base orientation condition and the right central grating as more vertical than the left one when both gratings are matched in orientation in the +4° base orientation condition. We speculate that this pattern could reflect a selection bias that may emerge in high uncertainty conditions^28^. Importantly, assuming that these biases are present in each condition, our estimates of contextual modulation strength would account for these biases given that we quantify the tilt effect by relative PSE differences in the T, V and VT conditions with respect to the N baseline condition.

Although we replicated the tilt illusion with a visual surround and observed significant contextual modulation effects, the size of the tilt illusion in our study was relatively small (~0.65°) compared to previous reports of tilt illusion effects (~2°)^5,23,24^. A number of methodological factors could account for these differences. First, in order to promote integration of the visual and tactile surrounds^19^, we corrupted the visual gratings in the surround using a high level of noise (96%). Because the central gratings contained noticeably less noise (50%), weaker contextual modulation could have been due to the disparity in noise levels as large differences between the central and surround gratings can drastically reduce the effectiveness of the surrounds to induce a strong tilt illusion effect^29,30^. Second, in order to match the motor demands across surround conditions, participants were required to perform a point-and-touch movement on each trial even when no orientation information was presented in the surround. The cognitive load imposed by these motor demands may have weakened contextual modulation effects. Lastly, the visual tilt illusion on visual-only trials may have been weak simply because subjects felt a smooth surface during the point-and-touch movements. Conceivably, incongruences between the visual and tactile cues in the surround may have attenuated the surround’s orientation signal strength thereby weakening its modulatory effect on the central grating.

Could this haptic-based enhancement of the visual tilt be due to an effect of attention, which can itself modulate visual contrast sensitivity^31,32^. Participants could be more engaged with the task in those blocks where the haptic gratings were presented with the surrounds (perhaps automatically due to its novelty or sensory richness compared with a flat haptic stimulus). This difference in salience could have enhanced the perceived contrast of the surround stimuli increasing the size of the illusion. However, a simple attention-based account is unlikely because a difference in attention across conditions should result in modulation of the sensitivity parameter (JND). Nonetheless, the JND parameter did not differ across the 4 experimental conditions.

Our primary finding was that a combination of congruent visual and tactile surrounds enlarged contextual modulation effects compared to a surround containing only visual signals. While the mere experience of tactile gratings in the surround was insufficient to induce a significant tilt illusion, the addition of the tactile surround augmented the effect of a visual surround that already induced small but significant modulatory effects. Because we failed to observe a significant interaction effect between the visual and tactile surround conditions, the larger tilt illusion observed with the visuo-tactile surround potentially reflects the sum of visual surround influences and independent (and weak) tactile surround influences. Although the surround stimuli were not tested at the limits of tactile and visual spatial acuity where vision would clearly dominate touch^33^, the orientation information available in our paradigm through the visual surround was likely still superior to that provided by touch. Thus, it is not surprising that the visual surround would modulate perception of the central grating more than the tactile surround. The weaker effects of the tactile surround may be attributable to a few factors. First, even as we ensured that the tactile stimulus covered a region of space that overlapped with the visual surround, the spatial area actually palpated in the surround was substantially smaller than the total surface area of the visual surround. Increasing the contacted area of the tactile surround may lead to stronger touch-induced modulation. Similarly, enhancing the tactile orientation signal by increasing the “contrast” of the tactile gratings could result in stronger tilt effects. It is also possible that tactile orientation cues can only influence visual perception in some but not all contexts. Indeed, even though tactile orientation cues can selectively bias visual grating perception under states of binocular rivalry, pre-exposure to tactile orientation signals does not prime visual perception in these ambiguous states as effectively as pre-exposure to visual stimuli^17^.

In the exploratory analyses we found a main effect of surround condition on λ, a parameter corresponding to lapse rates at the upper bound of the range of orientation differences we tested. Specifically, λ was larger in those conditions where the surround significantly modulated the perception of the central grating (Fig. 2E; Visual-only and Visuo-tactile surround; no significant difference between these conditions). Post-hoc correlation analyses confirmed that λ was positively correlated with changes in PSE across subjects (Fig. S5): Larger λ values were associated with subjects who experienced stronger contextual modulation effects. The larger λ values reflect the heterogeneous effects of the surround orientations, which were fixed at −15° and 15°, on the different central grating orientations, which we systematically varied. Because the strength of the tilt illusion depends on the center-surround orientation differences, the central gratings tested in the maximum positive difference condition experienced more repulsion from the surrounds. Thus, the λ parameter captures warping of the psychometric function which was caused by the non-uniform contextual modulation effects of the surround gratings. Moreover, lapse parameters also capture perceptual differences at asymptotic orientation difference values (−6° and +6°), which might be modulated by the tilt illusion (Fig. 1B). A congruent although non-significant pattern can be observed for the γ parameter, for which the largest value also takes place at the Visuo-tactile surround condition (Fig. 2D).

Our results relate to a number of recent studies that show that tactile orientation signals interact with the visual perception of grating orientation, particularly when the visual system is confronted with noisy or ambiguous information. For instance, when visual gratings that differ in orientation are presented to each eye, subjects perceive a grating that alternates between the two orientations. Touching a tactile grating can disambiguate visual perception under this binocular rivalry state by prolonging the dominance period for the visual grating that matches the tactile grating orientation^8,17^ or by reducing the strength of rivalry suppression effects^34^. Such tactile influences on binocular rivalry occur with passive or active touch, and these effects can be highly sensitive to the spatial frequencies and relative locations of the tactile and visual cues^17^. Touch similarly facilitates processing of visual grating stimuli that are rendered invisible by continuous flash suppression in a manner that depends on spatial frequency and orientation^35^. Our results build on this extensive literature by showing that touch also engages with neural processing related to contextual modulation in the tilt illusion. This finding may relate to a recent study reporting that temporal adaptation effects in orientation perception transfer from vision to touch^36^.

The neural computations underlying visual tilt effects are assumed to take place early in the visual processing pathway, possibly in primary visual cortex^2,5,37–39^. Accordingly, our results would suggest that tactile signals access neural circuits involved in early stages of visual processing to influence contextual modulation of the visual gratings. This interpretation is consistent with the fact that touch influences competitive visual processes like binocular rivalry, which are also believed to result from neural interactions in early visual cortices^40^. Because tactile stimulation alone has been shown to drive responses at multiple levels of the visual cortical hierarchy in human neuroimaging studies^41–44^ and single unit recordings^45,46^, tactile influences on visual contextual modulation may result from feedback signals originating in higher-order association areas that support attentional selection or predictive coding^47^. Alternatively, because causal manipulations of visual cortex modulate tactile orientation perception^48,49^, tactile influences on visual contextual modulation may result from direct interactions with tactile signals that are explicitly represented in visual cortex. Although our study cannot inform whether touch influences the visual tilt effects by modulating distributed inter-areal connectivity^50^ or local neural interactions^5^, our findings provide another example of how touch and vision interact selectively in spatial form processing. Moreover, given the recent interest in how visual contextual processing may be atypical in neurodevelopmental disorders like autism spectrum disorder and schizophrenia^51–53^, our study offers a novel approach for probing multisensory interactions and sensory processing in clinical and neurotypical populations.

## Methods

### Participants

Eighteen participants (12 male, 6 female) naïve to the experiment’s purpose (mean age = 21.4 years) were tested in this experiment. Written informed consent was obtained from all participants prior to testing. All participants reported normal or corrected-to-normal vision and normal tactile sensibilities. Subjects were predominantly right-handed (mean score = 69) according to Edinburgh handedness inventory scores, and we confirmed that the strength of tilt illusions induced under the Visuo-tactile condition did not relate statistically to hand dominance (R = 0.15, P = 0.4; see supplemental materials). The study was carried out in accordance with Declaration of Helsinki, under a protocol approved by the Institutional Review Board at Baylor College of Medicine, and the experiment was conducted in compliance with these guidelines. Participants were compensated $10 per hour for the duration of the experiment.

### Apparatus

Stimuli were displayed using a custom program in MATLAB utilizing Psychtoolbox-3. Visual stimuli were presented on a 27-inch Apple MC914LL/B display (resolution, 2560 × 1440) producing 375 cd/m^2^ peak brightness. Subjects responded using a dual-response foot pedal. Subjects sat at a fixed 50-cm distance from the display. The experiment took place in a windowless, dark room.

#### A bias-free measure of the tilt illusion

In order to obtain a bias-free measure of the tilt illusion, we implemented a simplified version of the paradigm used by^24,25^. In a two alternative forced choice (2AFC) paradigm, the participants had to simultaneously compare the orientation of two central gratings presented at both sides of the screen (Fig. 1). Both central gratings were surrounded by contextual gratings with opposite but symmetrical orientations. The participant’s goal was to judge which of the two central gratings was more closely oriented to the (subjective) vertical. The relative orientation difference between the two central gratings was varied in a trial-by-trial basis, always with respect to one of two reference base orientations (−4°, 4°). The contextual surround stimulus could contain just visual noise with no orientation information (“none” condition; N), visual noise with visual orientation information (Visual-only condition; V), visual noise with tactile orientation information (Tactile-only condition; T), or visual noise with congruent visual and tactile orientation information (Visuo-tactile condition; VT). The orientation of the contextual stimuli was kept constant during the whole experiment.

### Stimuli

The experimental display consisted of two visual stimuli vertically aligned and positioned 5 cm left and right of a central fixation point. These visual stimuli were center-surround sine-wave modulated full contrast gratings (spatial frequency = 1.6 degrees per cycle) that were combined with set percentage levels of static noise to reduce signal strength. The center component of each visual stimulus had a radius of 1.3 cm while the surround component had a radius of 2.6 cm. Tactile stimuli were circular pieces of flat (control) or ridged plastic approximately 6.5 mm in radius which were spatially overlaid on the surround portions of the visual gratings (Fig. 1C). The tactile grating stimulus (grating height: 1.5 mm; wavelength: 8 mm) had a spatial frequency and phase approximately matching the visual surround grating. Accordingly, the tactile grating stimulus spanned 2 full cycles. The tactile stimuli were a solid dark color which prevented subjects from seeing the tactile and visual gratings in the overlapping surround region. Stimuli orientations were determined on the following basis. The central gratings orientations were computed in each trial by combining one base and one relative orientation difference (*base orientation – orientation difference* for the left stimulus and *base orientation + orientation difference* for the right stimulus). Possible base orientations were ±4°, and possible relative orientation differences were ±0°, 1°, 3° or 6° (all degree measurements refer to the angle from vertical). The visibility of the central gratings was reduced by combining them with a 50% static white noise (50% signal). The surround grating orientations were fixed at −15° and +15° on the left and right visual stimuli respectively during the whole experiment. The surround visual noise was modulated depending on block condition. In the None and Tactile-only blocks, the surround pattern was 100% static white noise (0% signal), blocking all visual orientation information. In the Visual-only and Visuo-tactile conditions, surround patterns were displayed with a 96% noise (4% signal) overlay designed after pilot experiments to reproduce a reduced-magnitude tilt-effect. We aimed for a reduced-magnitude visual tilt-effect to prevent ceiling effects on the visual illusion and allow space for integrative effects between the visual and tactile surrounds. The ridged tactile stimuli used in the Visuo-tactile condition were oriented congruently to the surround of the visual stimuli on which they were placed: −15° on the left and +15° on the right. In the Visual-only and None conditions, flat tactile (control) stimuli that presented no orientation information were used instead.

### Procedure

The four different blocked conditions (N, V, T, and VT) were divided into eight blocks (two of each block condition) presented in pseudo-random order (each block condition had to be run once before it could be presented a second time). The experiment consisted of 1680 trials per participant (30 trials per condition, base orientation and orientation difference combination). At the beginning of each block, the subject was asked to look away while the experimenter placed the tactile stimuli required for the block, either the flat or the ridged tactile gratings, at their proper positions on the screen. The tactile stimuli were always placed in the same region of the visual inducers (Fig. 1C). Each trial began with a brief low-frequency auditory tone (340 Hz, 0.3 s) signaling the beginning of the trial. Subjects were instructed to maintain their gaze on a central fixation point and place their index fingers on the tactile stimuli when the auditory cue was presented. Participants were also instructed to touch the stimuli using their index fingers without performing exploring movements. The experimenter visually monitored participants’ compliance with these task instructions throughout the experiment. Then, the surround stimuli were displayed with the centers temporarily at 100% noise. After 1 s, the same surrounds but with the centers at only 50% noise were displayed for 0.4 s, followed by reversion to the 100% noise center for a further 0.5 s. Then, all visual stimuli disappeared, and a high-frequency auditory tone (440 Hz, 0.3 s) was played, signaling the subjects to remove their index fingers from the tactile stimuli. Subjects were then presented with a response screen with an “L” presented left of fixation and “R” presented right of fixation, prompting the subject to give their answer using the foot pedal. This response period lasted 2 seconds, after which if a response was not given, the response was skipped and the next trial began after a brief missed-response auditory tone. Before the experiment, subjects underwent a 15-min training period and were allowed to practice the task until they were comfortable with how to select the correct response and with synchronizing index finger placement and removal on each trial with the auditory cues. An experimenter sat close to the participant during the experiment to supervise their performance. Subjects were informed prior that their task exclusively concerned the orientations of the centers of the visual stimuli. The total duration of the experiment was 4 hours, divided in two days. Before the beginning of each block, the subject could take a short break of ~5 minutes.

### Data analysis

To estimate the magnitude of the tilt illusion, we used a parameter-as-outcome model (PAOM) method based on group-level statistical tests of psychophysical parameters estimated separately for each participant. To quantify each participant’s ability to discriminate the central grating orientation, we computed the proportion of trials in which the participant selected the right center grating as being closer to the vertical for each orientation difference, base orientation and experimental condition (N, T, V, VT). Then, we fitted the participant’s performance data using a cumulative Gaussian (eq. 1) with lapses (eq. 2)^54^:

1 $$F(x;\mu ,{\rm{\sigma }})=p({O}_{R} > {O}_{L})=\frac{1}{2}[1+{\rm{erf}}(\frac{{O}_{diff}-\mu }{{\rm{\sigma }}\sqrt{2}})]$$

2 $$\psi (x;\mu ,{\rm{\sigma }},\gamma ,\lambda )=\gamma +(1-\gamma +\lambda )F(x;\mu ,{\rm{\sigma }})$$

Using a non-linear optimization (the Nelder-Mead method implemented in R, *“optim”* function) we estimated the four free parameters (goodness of fit R^2^ = 0.990): μ is the point of subjective equality (PSE; the specific orientation difference in which the two central gratings are subjectively perceived equally close to the vertical) and σ is the slope of the function. The γ and λ lapse parameters are of no interest for testing our predictions, however fitting the logistic function with these two parameters allows a more accurate estimation of the PSE and JND parameters^54^. The γ parameter represents the distance of the left asymptote to 0 (lower bound), and the λ parameter represents the distance of the right asymptote to 1 (upper bound). We used the σ parameter to calculate the just noticeable difference (JND; i.e., the minimum difference in degrees between the two center gratings that a person can discriminate 50% of the time), which in this task is inversely related to the observer’s sensory precision in discriminating orientation information.

Because the two base conditions are symmetrically oriented with respect to the vertical (−4°, 4°), fitting the cumulative Gaussian functions to the data yield symmetric curves in both base orientation conditions (see Fig. S1). We re-oriented the data in the 4° base condition and −4° base condition by applying a simple transformation to the proportion values (proportion of left grating responses = 1-proportion of right grating responses). Re-orienting the data facilitates visual inspection of the effects in both base orientations while preserves the directionality of the PSE effects across the different experimental manipulations (Fig. S2).

In the group-level analysis, we first used ANOVA and t-tests to explore whether the average PSE, JND, γ and λ parameter estimates differed significantly as a function of experimental condition and base orientation (*Exploratory analyses section*). We determined whether any of the four estimated parameters for each experimental manipulation varied according to base orientation. Because we did not find any interaction between base orientation and surround condition (see Exploratory analyses), we re-fitted the model over the averaged proportions in both base orientations (Fig. S3). Modeling the data on the averaged probabilities improved the goodness of fit (R^2^ = 0.994) and reduced the model complexity [Bayesian information criterion (BIC) of the model with averaged base orientations = −25.5; BIC of the model with two base orientations = −21.5], favoring a more robust estimate of our parameters of interest. Second, in order to test our experimental predictions we performed repeated-measures ANOVA and t-tests on our parameter of interest (PSE) estimated from the data modeled after averaging the two base orientations together (*Hypotheses driven analyses section*). As we had clear predictions for how the presence of the surround gratings would modulate perception of the central gratings, we conducted one tailed t-tests. We reproduced our results in a complementary analysis using the PSE parameters estimated from the two base orientations modeled independently (see supplemental materials; Fig. S2). We also reproduced our results when analyzing PSE values estimated from psychometric functions that comprised a single parameter capturing the lower and upper asymptotes (see supplemental materials; Fig. S4).

## Electronic supplementary material

Supplemental materials
