# Supplementary material for: Touch engages visual spatial contextual processing
Source: Sci Rep. 2018 Nov 9;8:16637. doi: 10.1038/s41598-018-34810-z (PMC6226493; doi:10.1038/s41598-018-34810-z)
Supplement: Supplementary file 1 — Supplemental materials [file 41598_2018_34810_MOESM1_ESM.docx]

**Supplemental materials**

Touch engages visual spatial contextual processing

Alexis Pérez-Bellido, Ryan D. Pappal, Jeffrey M. Yau

*Hypothesis driven analyses on both base orientations fitted independently*

*1 Tilt illusion with visual surround*

In order to assess if the visual-only surrounds induced a visual tilt illusion we tested whether the PSE in the V condition was significantly larger than the PSE in the N condition. A one tailed paired t-test showed that the PSE in the V condition was 0.65^o^ larger than in the B condition (t_(17)_ = 5.518, P < 0.001, Cohen's d = 1.47). This result demonstrates that the surrounds effectively biased inward the subjective orientation percepts in both central gratings, and therefore an increment in their orientation difference (positive shift in the PSE) is required to counteract the visual tilt illusion effect.

*2 Tilt illusion with tactile surround*

Next, we tested whether a tactile surround alone is sufficient to induce the visual tilt illusion. A one tailed paired t-test contrasting whether the PSE in the T condition was larger than in the B condition showed that the direction of the effect matched our prediction (0.05^o^), but the difference was not significant (t_(17)_ = 0.67, P = 0.25). This result is inconclusive about whether tactile information is sufficient to induce the tilt illusion.

*3 Tilt illusion with visuo-tactile surround*

We investigated if combining visual and tactile surrounds enlarges the size of the tilt illusion. To do so, we tested whether the PSE in the VT condition was larger than in the V condition. A one tailed paired t-test showed that the PSE in the VT condition was 0.15^o^ larger than in the V condition (t_(17)_ = 1.8, P = 0.044, Cohen's d = 0.29).

*Hypothesis driven analyses modeling only one lapse parameter*

*1 Tilt illusion with visual surround*

In order to assess if the visual-only surrounds induced a visual tilt illusion we tested whether the PSE in the V condition was significantly larger than the PSE in the N condition. A one tailed paired t-test showed that the PSE in the V condition was 0.65^o^ larger than in the B condition (t_(17)_ = 5.40, P < 0.001, Cohen's d = 1.35). This result demonstrates that the surrounds effectively biased inward the subjective orientation percepts in both central gratings, and therefore an increment in their orientation difference (positive shift in the PSE) is required to counteract the visual tilt illusion effect.

*2 Tilt illusion with tactile surround*

Next, we tested whether a tactile surround alone is sufficient to induce the visual tilt illusion. A one tailed paired t-test contrasting whether the PSE in the T condition was larger than in the B condition showed that the direction of the effect matched our prediction (0.09^o^), but the difference was not significant (t_(17)_ = 1.16, P = 0.13). This result is inconclusive about whether tactile information is sufficient to induce the tilt illusion.

*3 Tilt illusion with visuo-tactile surround*

We investigated if combining visual and tactile surrounds enlarges the size of the tilt illusion. To do so, we tested whether the PSE in the VT condition was larger than in the V condition. A one tailed paired t-test showed that the PSE in the VT condition was 0.16^o^ larger than in the V condition (t_(17)_ = 2.12, P = 0.024, Cohen's d = 0.31).

*Hand dominance and size of the VT tilt illusion effect*

Finally, we tested whether differences in hand dominance correlated with the size of the Visuo-tactile tilt illusion. We calculated the Visuo-tactile PSE bias for each participant and base orientation condition by subtracting the PSE in the V condition to the PSE in the VT condition. Then, we calculated the Pearson correlation coefficient for the Visuo-tactile PSE bias with the Edinburgh handedness inventory scores in each base orientation condition. We did not find significant correlations for the -4^o^ base orientation (R = 0.05, P = 0.85) nor for the 4^o^ (R = -0.13, P = 0.62). A similar analysis was performed on the PSE parameters obtained after fitting the psychometric functions on the averaged base orientation conditions leading to identical results (R = 0.15, P = 0.4). Thus, we can conclude that hand dominance did not determine or biased the strength of the visuo-tactile tilt illusion effect in our experiment.


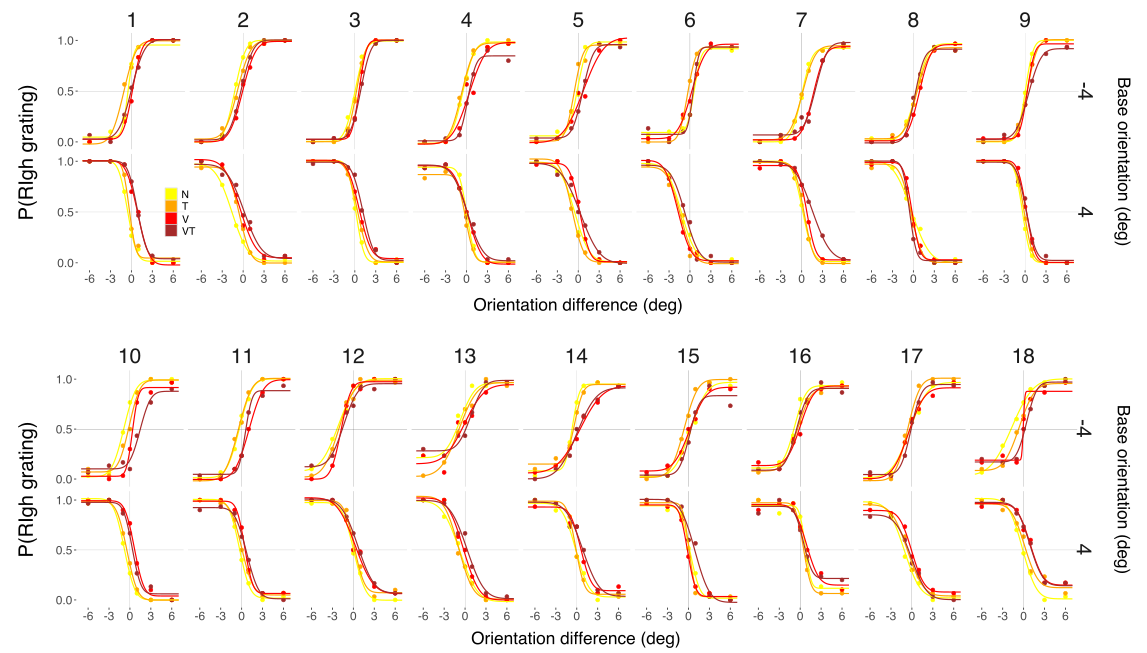


**Figure S1 Individual fits** Points represent orientation discrimination performances in both base orientation conditions. Lines represent the fitted psychometric functions for each participant, experimental condition and base orientation.


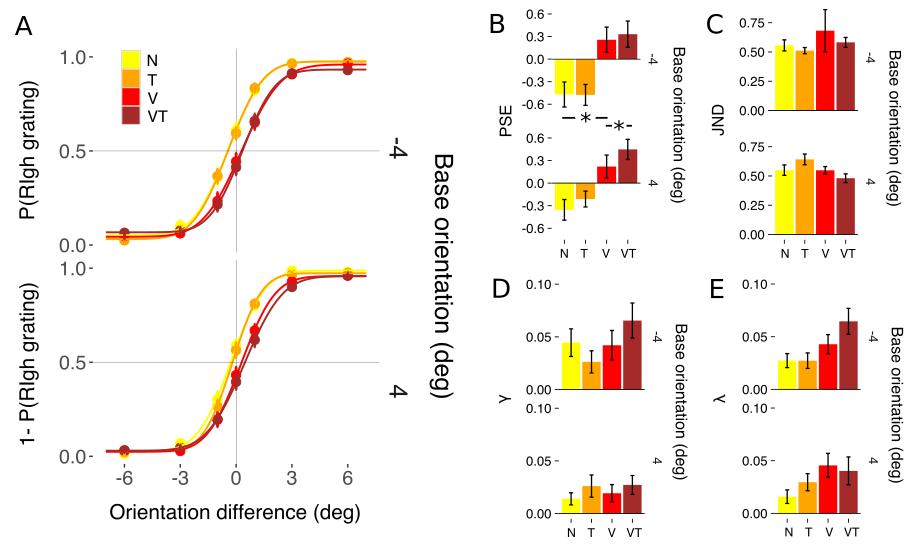


**Figure S2 Orientation discrimination performances modeling both base orientation conditions**. Proportion data in base orientation 4^o^ have been re-oriented to match base orientation -4^o^. (A) Group average proportion data and group level psychometric functions for each condition. Mean of the group distribution of individual PSE (B), JND (C), γ (D) and λ (E) parameters. Error bars represent standard error. Asterisks represent those hypothesis driven contrasts that showed a significant effect.


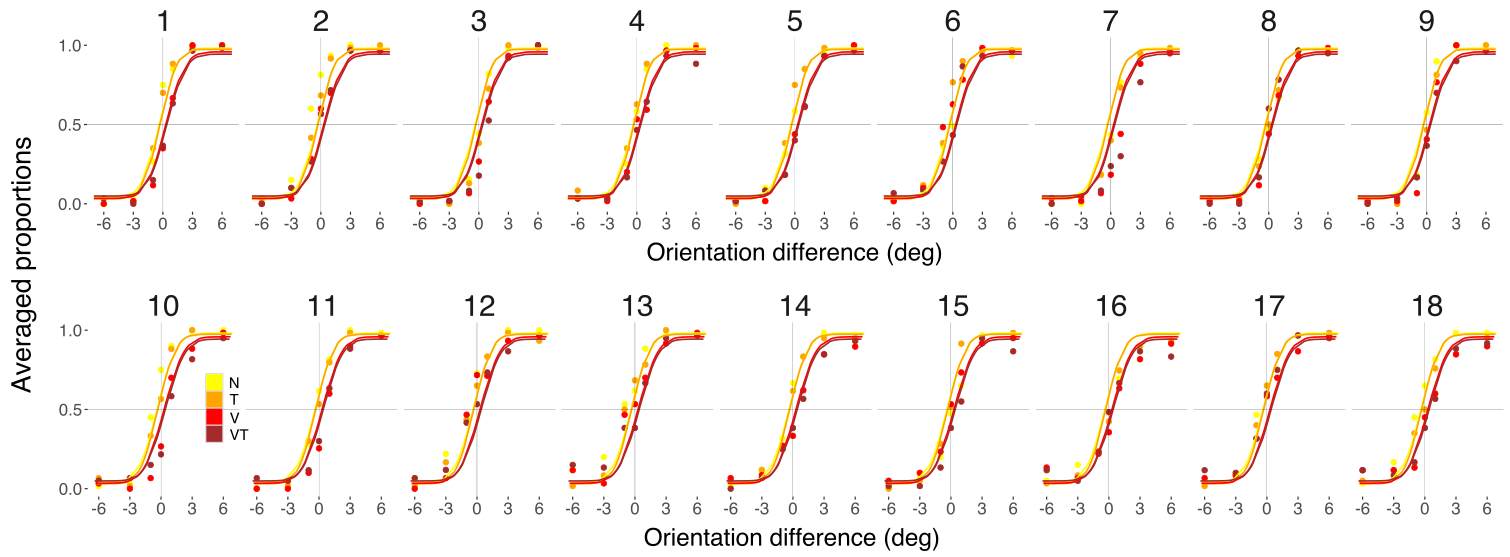


**Figure S3 Individual fits** Points represent orientation discrimination performances after averaging the proportion of responses in both base orientation conditions. Note that proportions in base orientation condition 4^o^ were re-oriented prior to be averaged with the -4^o^ condition. The lines depicted in each panel represent the fitted psychometric functions to the averaged proportions for each participant, experimental condition and base orientation.


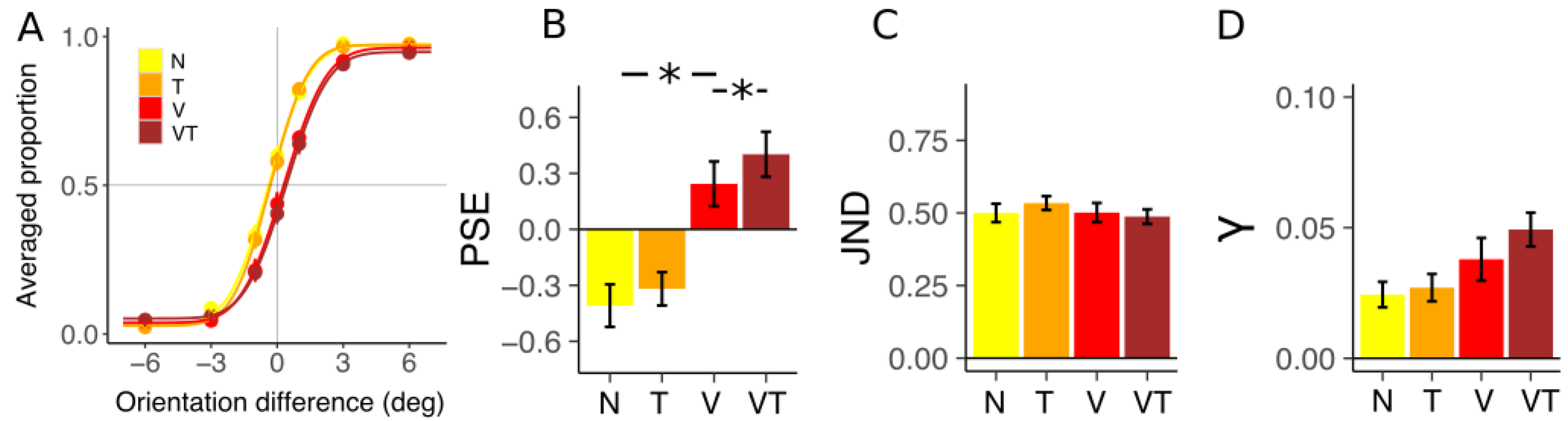


**Figure S4 Orientation discrimination performance modeling only one (γ) lapse parameter** **A:** Group averaged choice probability data and group level psychometric functions for each surround condition (None, Tactile-only, Visual-only and Visuo-tactile). Mean of the group distribution of individual PSE (***B***), JND (***C***), γ (***D***) parameters. Error bars represent standard error. Asterisks represent those hypothesis-driven contrasts that showed a significant effect.


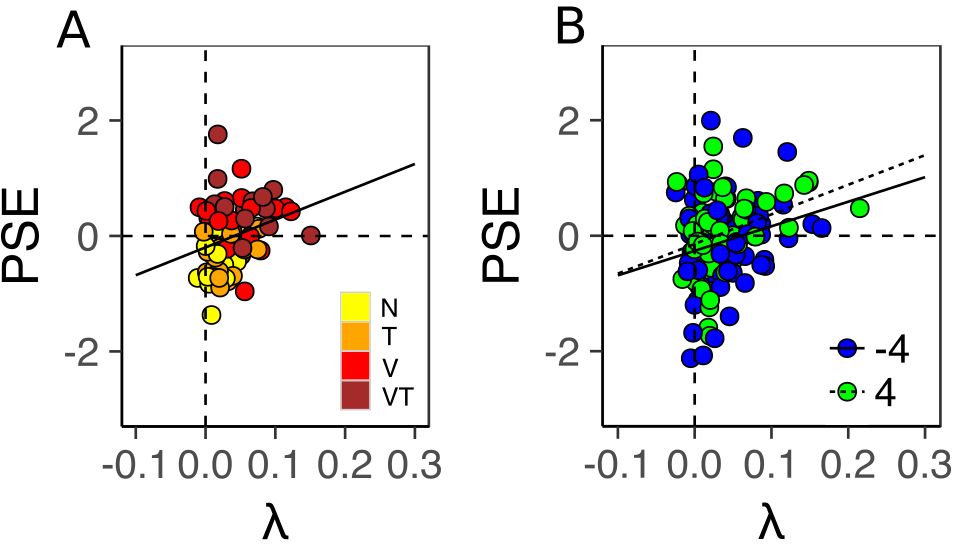


**Figure S5 Correlation between the PSE and λ parameters** (A) Each point represents an individual PSE parameter as a function of its corresponding λ in each surround condition. The black line represents the linear regression fit (R = 0.29, p = 0.014). (B) Same data as in A but dividing the analyses by base orientation condition. Correlation coefficients for the -4^o^ base orientation condition (R = 0.22, p = 0.058) and 4^o^ base orientation condition (R = 0.35, p < 0.003).
